# Comparison of neoadjuvant chemohormonal therapy vs. extended pelvic lymph-node dissection in high-risk prostate cancer treated with robot-assisted radical prostatectomy

**DOI:** 10.1038/s41598-023-30627-7

**Published:** 2023-03-01

**Authors:** Takuya Oishi, Shingo Hatakeyama, Ryuji Tabata, Daiji Fujimori, Mamoru Fukuda, Tetsuo Shinozaki, Noritaka Ishii, Hiromichi Iwamura, Teppei Okamoto, Hayato Yamamoto, Takahiro Yoneyama, Yasuhiro Hashimoto, Satoshi Sato, Chikara Ohyama

**Affiliations:** 1 Department of Urology, Hirosaki University Graduate School of Medicine, Hirosaki, Japan; 2 Department of Advanced Blood Purification Therapy, Hirosaki University Graduate School of Medicine, 5 Zaifu-Chou, Hirosaki, 036-8562 Japan; 3 Department of Urology, Ageo Central General Hospital, Ageo, Japan; 4 Department of Advanced Transplant and Regenerative Medicine, Hirosaki University Graduate School of Medicine, Hirosaki, Japan

**Keywords:** Cancer, Medical research, Oncology, Urology, Prostate

## Abstract

We compared the impact of treatment strategies on postoperative complications and prognosis between robot-assisted radical prostatectomy (RARP) plus extended pelvic lymph-node dissection (ePLND) and RARP plus neoadjuvant chemohormonal therapy (NCHT) without ePLND. We retrospectively evaluated 452 patients with high-risk prostate cancer (defined as any one of prostate-specific antigen ≥ 20 ng/mL, Gleason score 8–10, or cT2c–3) who were treated with RARP between January 2012 and February 2021. The patients were divided into two groups: RARP with ePLND (ePLND group) and NCHT plus RARP without ePLND (NCHT group). We compared the complication rate (Clavien–Dindo classification), biochemical recurrence-free survival, and castration-resistant prostate cancer (CRPC)-free survival between the groups. We performed multivariable Cox regression analysis using inverse probability weighting (IPTW) methods to assess the impact of the different treatments on prognosis. There were 150 and 302 patients in the ePLND and NCHT groups, respectively. The postoperative complication rate was significantly higher in the ePLND group than in the NCHT group (*P* < 0.001). IPTW-adjusted biochemical recurrence-free survival and CRPC-free survival were significantly higher in the NCHT group than in the ePLND group (hazard ratio [HR] 0.29, *P* < 0.001, and HR 0.29, *P* = 0.010, respectively). NCHT plus RARP without ePLND may reduce the risk of postoperative complications compared with ePLND during RARP. The impact of treatment strategies on oncological outcomes needs further studies.

## Introduction

Prostate cancer (PC) is the most common malignancy in men in Western countries and in Japan. Although radical prostatectomy (RP) is one of the standards of care in localized PC^1^, the optimal treatment for high-risk PC remains unclear. The current European Association of Urology (EAU) guidelines recommend extended pelvic lymph-node dissection (ePLND) for intermediate- and high-risk disease for optimal staging^2^, while those of the American Urological Association (AUA) do not make any recommendation for ePLND because the evidence supporting its therapeutic benefit is lacking^3^. Recently, two randomized controlled trials compared extended and limited PLND. They found that ePLND did not improve biochemical recurrence-free survival (BCR-FS) compared with limited PLND^4,5^. Furthermore, ePLND may increase postoperative complications. Therefore, the indications for ePLND, the optimal extent of lymph-node dissection, and the balance between the benefits and harms of treatment must be carefully considered^6,7^.

Neoadjuvant hormonal therapy (NHT) followed by RP is an alternative option that uses androgen deprivation therapy (ADT) or ADT plus bicalutamide to treat high-risk PC. However, there is insufficient evidence to assess the possible effect of NHT in cases of high-risk PC^8–10^. Our previous study on patients with high-risk PC demonstrated that neoadjuvant chemohormonal therapy (NCHT) plus open RP using ADT and low-dose estramustine phosphate (EMP) significantly improved BCR-FS compared with patients treated with open RP and ePLND^11^. Thus, an alternative option might be NCHT using ADT plus a low dose of EMP. However, the oncological outcomes of robot-assisted radical prostatectomy (RARP) plus ePLND versus NCHT plus RARP without ePLND have yet to be compared. To this end, we compared the impact of treatment strategies on postoperative complications and oncological outcomes between ePLND (ePLND group) and NCHT without ePLND (NCHT group) in patients with high-risk PC treated with RARP.

## Results

### Baseline characteristics

**Figure 1:**
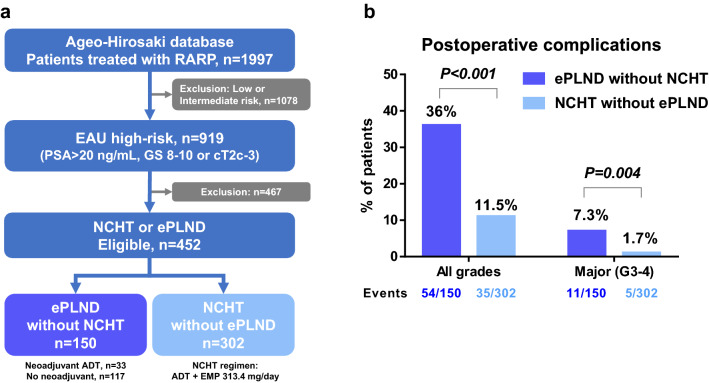
Patient selection and postoperative complications. (**a**) Patient selection. (**b**) Comparison of postoperative complications. *ADT* androgen deprivation therapy, *EAU* European Association of Urology, *EMP* estramustine phosphate, *ePLND* extended pelvic lymph-node dissection, *GS* Gleason score, *NCHT* neoadjuvant chemohormonal therapy, *PSA* prostate-specific antigen, *RARP* robot-assisted radical prostatectomy.

We used the Ageo-Hirosaki database to retrospectively identify 1997 patients treated with RARP, of whom 919 were identified as PC patients with high-risk disease. After applying the exclusion criteria, there were 150 and 302 patients in the ePLND and NCHT groups, respectively (Fig. 1a). The median age and follow-up periods were 69 (IQR 65, 72) years old and 62 (IQR 41, 88) months, respectively, in this cohort. There was a significant difference between the ePLND and NCHT groups in PSA, Gleason score, and clinical T stage at baseline (Table 1). The median duration of NCHT in the NCHT group was 8.7 (IQR 7.1, 10) months. In the ePLND group, 33 (22%) patients underwent NHT with either ADT alone or ADT plus bicalutamide (Table 1). The median risk of lymph-node invasion in the NCHT and ePLND groups was 16% (IQR 8%, 38%) and 41% (IQR 20%, 70%) respectively (P < 0.001). as determined by the Briganti nomogram^12^.

**Table 1:** Background of patients.

	ePLND	NCHT	P value
n	150	302	
Age, years (IQR)	70 (66, 72)	68 (65, 72)	0.226
Age > 75 years, n	15 (10%)	28 (9.3%)	0.865
ECOG PS > 0, n	3 (2.0%)	2 (0.7%)	0.338
PSA, ng/mL	12.3 (6.9, 22.8)	9.6 (6.3, 10.3)	0.002
PSA ≥ 20 ng/mL, n	31 (20.7%)	69 (22.8%)	0.632
GS, n			0.006*
6 or 7	27 (18.0%)	27 (8.9%)	
8	26 (17.3%)	90 (29.8%)	
9 or 10	97 (64.7%)	185 (61.3%)	
% of positive core	50% (33, 75)	40% (25, 50)	< 0.001
Clinical T stage, n			0.001**
cT1	1 (0.7%)	101 (33.4%)	
cT2	66 (44.0%)	89 (29.5%)	
cT3	77 (51.3%)	112 (37.1%)	
Neoadjuvant hormonal therapy, n	33 (22%)		
NCHT periods, months (IQR)		8.7 (7.1, 10)	
Limited PLND	0 (0%)	135 (44.7%)	
Surgical time, min (IQR)	289 (242, 341)	169 (147, 198)	< 0.001
Blood loss, g (IQR)	150 (100, 250)	25 (10, 50)	< 0.001
Pathological T, n			
pT0	1 (0.7%)	36 (11.9%)	< 0.001
pT2	61 (40.7%)	185 (61.3%)	< 0.001
pT3	84 (56.0%)	81 (26.8%)	< 0.001
pT4	4 (2.7%)	0 (0%)	0.012
Resection margin+, n	44 (29.3%)	26 (8.6%)	< 0.001
Pathological N+, n	43 (28.7%)	1 (0.3%)	< 0.001
Median follow-up, months (IQR)	41 (27, 58)	80 (56, 98)	
Biochemical recurrence (BCR), n	66 (44.0%)	51 (16.9%)	
CRPC progression, n	12 (8.0%)	9 (3.0%)	
Any cause of death, n	4 (2.7%)	4 (1.3%)	

*PSA* prostate-specific antigen, *ECOG PS* Eastern Cooperative Oncology Group performance status, *ePLND* extended pelvic lymph node dissection, *NCHT* neoadjuvant chemohormonal therapy.

*GS6-7 vs. 8–10, **cT1-2 vs. cT3.

### Surgical and pathological outcomes

The median operation time and blood loss were significantly different between the ePLND group (289 min and 150 g, respectively) and the NCHT group (169 min and 25 g, respectively). Additionally, the pathological tumor stage was also significantly different between the groups (Table 1). The median number of removed lymph-nodes and the rate of positive nodes in patients in the NCHT group who underwent limited PLND (n = 135/302, 44.7%) were 4 (IQR 3, 7) and 0.74% (n = 1/135), respectively. The median number of removed lymph-nodes and the rate of positive nodes in the ePLND group (n = 150) were 23 (IQR 17, 28) and 28.7% (n = 43/150), respectively.

The rate of postoperative complications of any grade and the rate of major complications in the ePLND group was significantly higher (36.0% and 7.3%, respectively) compared with the NCHT group (11.5% and 1.7%, respectively) (Fig. 1b, Table 2). The multivariable logistic regression analysis for any grade complications shows that ePLND is the independent factor for increased risk of postoperative major complications (OR 5.06, P < 0.001, Table 3).

**Table 2:** Summary of postoperative complications.

	ePLND	NCHT	P value
Postoperative complication (any grade), n	54 (36%)	35 (11.5%)	< 0.001
Major complication (grade 3 or more), n	11 (7.3%)	5 (1.7%)	< 0.001
Ileus	4 (2.7%)		
Inguinal hernia	1 (0.7%)		
Anastomotic leakage	1 (0.7%)		
Delirium	1 (0.7%)		
Missing clip	1 (0.7%)		
Lymphocele/lymphatic fistula	3 (2.0%)	1 (0.3%)	
Cerebral infarction		1 (0.3%)	
Cardiovascular event		1 (0.3%)	
Rectal injury		1 (0.3%)	
Ureteral stricture		1 (0.3%)

**Table 3:** Multivariable logistic regression analysis for postoperative complication.

		P value	Odds ratio	95% CI
Age	Continuous, years	0.943	1.00	0.96–1.05
Gleason score	9 or 10	0.922	1.00	0.99–1.01
cT stage	cT3	0.628	0.88	0.53–1.47
PSA	Continuous, ng/mL	0.578	0.87	0.52–1.44
Neoadjuvant ADT alone	Yes	0.178	0.53	0.21–1.34
ePLND	Yes	< 0.001	5.06	3.01–8.51

### Oncological outcomes

**Figure 2:**
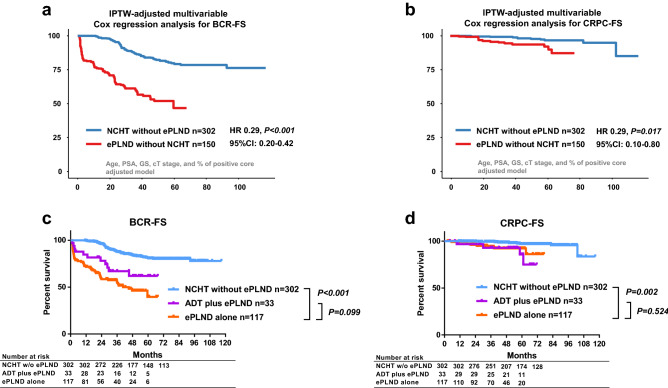
Comparison of oncological outcomes between ePLND during RARP and NCHT plus RARP without ePLND. (**a**) The Cox regression analysis using inverse probability weighting (IPTW) methods of biochemical recurrence-free survival (BCR-FS). (**b**) The Cox regression analysis using IPTW methods of castration-resistant prostate cancer-free survival (CRPC-FS). (**c**) Unadjusted analysis of biochemical recurrence-free survival (BCR-FS). (**d**) Unadjusted analysis of castration-resistant prostate cancer-free survival (CRPC-FS). *ePLND* extended pelvic lymph-node dissection, *GS* Gleason score, *NCHT* neoadjuvant chemohormonal therapy, *PSA* prostate-specific antigen, *RARP* robot-assisted radical prostatectomy.

The inverse probability weighting (IPTW)-adjusted analysis showed that BCR-FS was significantly lower in the ePLND group than that in the NCHT group (HR 0.29, *P* < 0.001, Fig. 2a). Similarly, the IPTW-adjusted castration-resistant prostate cancer-free survival (CRPC-FS) was significantly lower in the ePLND group than in the NCHT group (HR 0.29, *P* = 0.017, Fig. 2b).

The unadjusted BCR-FS rate was significantly higher in the patients with NCHT than those in the ePLND alone (*P* < 0.001, Fig. 2c). The unadjusted CRPC-FS rate was significantly higher in the patients with NCHT than those in the ePLND alone (*P* = 0.002, Fig. 2d). Oncological outcomes between treatment with ePLND alone (n = 117) and neoadjuvant ADT therapy plus ePLND (n = 33) were not significantly different for BCR-FS (Fig. 2c, P = 0.099) and CRPC-FS (Fig. 2d, P = 0.524).

### EMP-related toxicities

We evaluated 302 patients who were treated with neoadjuvant EMP + ADT in this study. Of those, we observed 58 (19.2%) patients with EMP-related toxicities. The major toxicity was low grade (grade 1–2) liver dysfunction (7.0%) followed by gastrointestinal symptoms (6.3%). We had severe cardiovascular events (suspicion of angina pectoris) in 1 patient (0.3%) that required short-term hospitalization (Table 4).

**Table 4:** EMP related adverse events (Neoadjuvant EMP, n = 302).

	All	G1	G2	G3
EMP-related AEs, n	58 (19.2%)	44 (14.6%)	13 (4.3%)	1 (0.3%)
Liver disfunction, n	21 (7.0%)	16 (5.3%)	5 (1.7%)	0 (0%)
Gastrointestinal symptoms, n	19 (6.3%)	18 (6.0%)	1 (0.3%)	0 (0%)
Cardiovascular events, n	3 (1.0%	0 (0%)	2 (0.7%)	1 (0.3%)
Skin rash, n	7 (2.3%)	5 (1.7%)	2 (0.7%)	0 (0%)
Edema, n	3 (1.0%)	1 (0.3%)	2 (0.7%)	0 (0%)
Anemia, n	3 (1.0%)	3 (1.0%)	0 (0%)	0 (0%)
Dysgeusia, n	1 (0.3%)	1 (0.3%)	0 (0%)	0 (0%)
Nipple pain, n	1 (0.3%)	1 (0.3%)	0 (0%)	0 (0%)

Furthermore, we additionally evaluated all patients (n = 533) who were treated with neoadjuvant EMP + ADT in our database. Of those, we observed 105 (19.7%) patients with EMP-related toxicities. The major toxicity was low grade (grade 1–2) gastrointestinal symptoms (7.3%) followed by liver dysfunction (6.2%). We had severe cardiovascular events in 3 patients (0.6%) that required short-term hospitalization (Table S1).

## Discussion

We compared the impact on prognosis between ePLND and NCHT (ADT + EMP) without ePLND in high-risk PC treated with RARP. NCHT without ePLND was significantly associated with a lower rate of postoperative complications (difference of 24.5% across all grades), prolonged BCR-FS, and prolonged CRPC-FS compared with ePLND. However, the analyses of CRPC progression were underpowered because of the small number of events (21 and 8 events, respectively). These limitations were imposed by the retrospective nature of our study. Furthermore, it is well-known that the role of NCHT in the context of PC is still highly debated and many prospective studies have failed to demonstrate its efficacy on oncological outcomes. Thus, our data should serve as a basis to perform a clinical trial and needs to be validated by prospective studies.

There have been numerous attempts to treat cancer using lymph-node dissection. However, several randomized controlled trials did not show any therapeutic benefits of PLND in bladder, esophageal, gastric, pancreatic, lung, breast, and ovarian cancers, except for colorectal cancer^13–20^. The benefit of PLND during RP had been discussed in retrospective studies, and it was reported that the diagnostic benefit of ePLND is its ability to identify twice as many nodal metastases as limited PLND and may help to cure nodal micrometastases^21–25^. The 2021 EAU guidelines strongly recommend ePLND for intermediate-risk disease with an estimated risk for positive lymph-nodes ≥ 5% and all high-risk diseases for optimal nodal staging^2^. Conversely, the 2017 AUA guidelines do not recommend ePLND because of the lack of evidence supporting its therapeutic benefit^3^. A recent systematic review including 44 retrospective studies (n = 275,269) found no significant difference in survival benefit between any form of PLND and no PLND^26^. Furthermore, two randomized controlled trials did not demonstrate survival benefits of ePLND compared with limited PLND^4,5^. Currently, there is no level 1 evidence confirming the survival benefit of ePLND. It is hoped that an ongoing trial comparing ePLND with no PLND (NCT03921996) will provide information on whether or not PLND can be omitted during RP.

It is unclear whether extended PLND provides an oncologic benefit over limited PLND^4,5^. A previous randomized study including 1440 patients (NCT01407263) suggested that extended PLND (the median number of nodes removed: 14, IQR 10–20) did not improve biochemical recurrence-free survival over limited PLND (the median number of nodes removed: 12, IQR 8–17) for men with clinically localized prostate cancer^5^. However, the number of lymph-nodes removed between the two groups in this study may be too close for a proper comparison. Furthermore, little is known about the significance of PLND in the case of neoadjuvant therapy. Some of the patients in the NCHT group underwent limited PLND in this study. Based on the results of our previous study^27^, we included both localized and no dissection in the neoadjuvant group. However, the mixture of limited and no PLND in the neoadjuvant group is a limitation of this study. The appropriate extent of dissection in neoadjuvant therapy needs further investigation.

Although there is insufficient evidence to show a survival benefit of NHT in the high-risk PC population^8–10^, a strategy of intensive therapy using second-generation androgen receptor axis-targeting agents (ARATs) might be reasonable for high-risk nonmetastatic PC instead of ePLND. A recent phase III randomized trial (STAMPEDE) showed a survival benefit of the combination of radiotherapy (99% and 71% of cN0 and cN1 patients received local radiotherapy, respectively) and ARATs (abiraterone acetate with or without enzalutamide) in men with nonmetastatic PC^28^. However, ARATs are not approved for nonmetastatic PC in Japan at present. Accordingly, our NCHT strategy for high-risk PC may be a promising option because of its shorter operating time and lower amount of blood loss, low rate of postoperative complications, and its contribution to survival. However, the toxicity of EMPs must be noted, particularly because it can cause liver dysfunction and gastrointestinal symptoms. We experienced discontinuations due to the grade 1 or 2 toxicity of EMP (313.4 mg/day) in 3.2% of cases (17/533) in the entire cohort. Although no thrombosis was observed in this study, thrombotic events with estrogenic agents are definitely a concern, even at half doses. Therefore, not all high-risk patients are candidates for NCHT using EMP because of the intensive efficacy of estrogen and nitrogen mustard. The ongoing phase III, randomized, double-blind, placebo-controlled, multicenter PROTEUS trial (NCT03767244)^29^ might change the treatment paradigm of high-risk PC because it is evaluating the efficacy (dual primary endpoints of pathological complete response rate and metastasis-free survival) and safety of apalutamide plus ADT compared with placebo plus ADT before and after RP in patients with localized or locally advanced high-risk PC. However, the results will only be available in 2027.

The present study has several limitations. First, the limited sample size, retrospective design, and background differences between groups prevent us from drawing a definitive conclusion. Second, the follow-up period was shorter in the ePLND group. Third, our results cannot be generalized to non-Asian populations because of racial differences. Finally, a direct comparison of NCHT with ePLND on oncological outcomes is inconclusive based on retrospective data. The statistical methodology (IPTW) could not resolve all unmeasured confounders and residual confounding may still play a role. Our data should serve as a basis to perform a clinical trial.

In conclusion, NCHT plus RARP without ePLND may reduce the risk of postoperative complications in comparison with ePLND during RARP. The impact of treatment strategies on oncological outcomes needs further prospective studies.

## Methods

### Ethics statement

This study was performed in accordance with the ethical standards established by the Declaration of Helsinki and was approved by the Ethics Review Board of Hirosaki University Graduate School of Medicine and all hospitals (authorization number: 2021-2419).

### Study design and participants

We used the Hirosaki and Ageo database to retrospectively evaluate 1997 patients with PC who underwent RARP at Hirosaki University Hospital and Ageo Central General Hospital between January 2012 and February 2021. Information on the patients’ background; disease status; and surgical, pathological, and oncological outcomes were obtained from their medical records. All tumors were staged according to the 2017 American Joint Committee on Cancer staging manual^30^. The inclusion criteria were as follows: (1) patients with a high-risk disease defined as any one of prostate-specific antigen (PSA) ≥ 20 ng/mL, a Gleason score 8–10, or cT2c–4; (2) clinically negative lymph-node metastases (cN0); and (3) patients who underwent ePLND without NCHT or NCHT without ePLND. The indication, duration, and type of NHT or NCHT depended on institutional protocols. The risk of lymph-node invasion was evaluated using the Briganti nomogram with a cutoff value of ≥ 5% in patients with ePLND^12^. The exclusion criteria were as follows: (1) patients who underwent open RP, (2) metastatic disease, (3) missing data on initial PSA level, biopsy Gleason score, and cT stage, and (4) missing survival data and/or follow-up duration.

### Treatment procedures

The RARP and ePLND were performed by expert surgeons. The ePLND template included the obturator, external iliac, and internal iliac regions bilaterally. The ePLND group included some patients treated with NHT plus ePLND for very high-risk disease. The patients in the NCHT group received ADT (luteinizing hormone-releasing hormone agonist or gonadotropin-releasing hormone antagonist) plus low-dose EMP (313.4 mg/day) for 6–9 months before RARP, as previously described^11^. The patients in the NCHT group underwent limited PLND (removal of the bilateral obturator node chains) or no PLND.

### Follow-up protocol

After surgery, the serum levels of PSA and testosterone were tested for all patients every 3 months. Adjuvant ADT or radiation therapy was not routinely administered. The date of BCR was defined as the date when the serum PSA level exceeded 0.2 ng/mL. If the PSA level did not decrease to < 0.2 ng/mL after the surgery, the date of RARP was defined as the date of BCR. Castration-resistant prostate cancer (CRPC) was defined by the Prostate Cancer Working Group 2 or identification of clinical progression by attending physician. CRPC-free survival (CRPC-FS) was defined from the time of surgery to the CRPC progression or any cause of death.

### Outcomes

We divided the patients into two groups: RARP with ePLND (ePLND group) and NCHT plus RARP without ePLND (NCHT group). We compared the complication rate (Clavien–Dindo classification), BCR-FS and CRPC-FS between the groups. We also assessed the impact of treatment on prognosis using multivariable Cox regression analysis via inverse probability weighting (IPTW) methods. Overall survival was not compared between the groups due to the very small events (n = 8).

### EMP-related toxicities

We evaluated EMP-related toxicities to estimate the balance between benefit and harm. We included all patients who were treated with neoadjuvant EMP + ADT in our database (n = 533). Toxicity was evaluated using Common Terminology Criteria for Adverse Events ver. 5.0.

### Statistical analysis

Statistical analyses were performed using the Microsoft Excel (Microsoft Corp., Redmond, WA, USA), Bell curve in Excel (Social Survey Research Information Co., Ltd., Tokyo, Japan) and GraphPad Prism 7.00 (GraphPad Software, San Diego, CA, USA) and R v4.0.2 (The R Foundation for Statistical Computing, Vienna, Austria) software. Categorical variables were compared using Fisher exact test or χ^2^ test. Quantitative variables were expressed as medians with interquartile ranges (IQRs). The differences between the groups were compared using Student *t*-test or Mann–Whitney U test. Odds ratio (OR) with 95% confidence interval (95% CI) were obtained from the multivariable logistic regression analysis for any grade postoperative complications. BCR-FS and CRPC-FS were compared using the Kaplan–Meier method. Hazard ratios (HRs) with 95%CI were obtained from the multivariable Cox regression analysis using PTW methods. The variables included in the IPTW-adjusted model were age (continuous), PSA (continuous), GS (6–10), cT stage (1–3), and % of positive core (7–100%). *P* values < 0.05 were considered statistically significant.

### Ethical approval

This retrospective, multicenter study was performed per the ethical standards of the Declaration of Helsinki and was approved by the Ethics Review Board of Hirosaki University School of Medicine (authorization No. 2021-2419) and all hospitals.

### Patient consent statement

An informed consent was obtained from all participants via written, verbal, and/or disclosure of study information.

## Supplementary Information

Supplementary Table S1.

## Data Availability

Data are available for bona fide researchers who request it from the authors.
