# Supplementary material for: Comparison of neoadjuvant chemohormonal therapy vs. extended pelvic lymph-node dissection in high-risk prostate cancer treated with robot-assisted radical prostatectomy
Source: Sci Rep. 2023 Mar 1;13:3436. doi: 10.1038/s41598-023-30627-7 (PMC9978020; doi:10.1038/s41598-023-30627-7)
Supplement: Supplementary file 1 — Supplementary Table S1. [file 41598_2023_30627_MOESM1_ESM.docx]

**Table S1; EMP related adverse events (All patients with neoadjuvant ADT + EMP n = 533)**

|  | All | Grade 1 | Grade 2 | Grade 3 |
| --- | --- | --- | --- | --- |
| EMP-related AEs, n | 105 (19.7%) | 69 (12.9%) | 33 (6.2%) | 3 (0.6%) |
| Gastrointestinal symptoms, n | 39 (7.3%) | 34 (6.4%) | 5 (0.9%) |  |
| Liver disfunction, n | 33 (6.2%) | 22 (4.1%) | 11 (2.1%) |  |
| Cardiovascular events, n | 11 (2.1%) |  | 8 (1.5%) | 3 (0.6%) |
| Skin rash, n | 11 (2.1%) | 7 (1.3%) | 4 (0.8%) |  |
| Edema, n | 4 (0.8%) | 1 (0.2%) | 3 (0.6%) |  |
| Anemia, n | 3 (0.6%) | 3 (0.6%) |  |  |
| Deep vein thrombus, n | 1 (0.2%) |  | 1 (0.2%) |  |
| Hypercortisolemia, n | 1 (0.2%) |  | 1 (0.2%) |  |
| Dysgeusia, n | 1 (0.2%) | 1 (0.2%) |  |  |
| Nipple pain, n | 1 (0.2%) | 1 (0.2%) |  |  |
